# Evidence of Negative Effects of Defect Size and Older Patient Age by Quantitative CT-Based 3D Image Analysis in Ultraporous Beta-Tricalcium Phosphate Grafted Extremity Bone Defects at One Year

**DOI:** 10.1155/2018/5304215

**Published:** 2018-11-01

**Authors:** Timothy A. Damron, Kenneth A. Mann

**Affiliations:** SUNY Upstate Medical University, Syracuse, New York, USA

## Abstract

**Introduction:**

Synthetic bone graft materials are commonly used to fill defects after curettage of benign bone lesions. Ultraporous beta tricalcium phosphate (TCP) is a popular synthetic compound used in this situation. Prior clinical studies based on plain X-ray analysis suggest incorporation of TCP is incomplete, even when combined with bone marrow (BMA).

**Purpose:**

The purpose was to analyze volumetric CT-based changes in defects grafted with TCP with/without BMA in a completed prospective RCT to objectively determine (1) relationship between size and age versus TCP incorporation and (2) whether there is an advantage to addition of BMA.

**Methods:**

Twenty-one patients with CT scans at ≥1 year follow-up available for digital analysis (TCP=10, TCP w/BMA =11) form the study population. CT image stacks were evaluated by creating volumetric masks using MIMICS imaging software for total defect, graft remaining, and graft incorporated volumes graft incorporation endpoints.

**Results:**

Overall, there was significant (p=0.0029) negative correlation (r^2^ = 0.38) between defect size and ratio of incorporated bone to defect size. This relationship remained strong (r^2^ = 0.56) particularly for defects > 20 cc but not for smaller defects. Bone width was also a significantly related factor (r^2^ = 0.94), with less graft incorporation in larger bone sites, in part likely due to the linear relationship between defect size and bone width. Relationship with age was complex and closely tied to defect volume. For larger defect volumes, younger patients were more successful at graft incorporation. Although age itself was not an independently significant factor, as defect volume increased, advanced age more negatively impacted new bone formation.

**Conclusions:**

Larger size defect and affected bone and advancing age appear to be important negative factors in synthetic graft incorporation. Results showed no advantage to addition of BMA to TCP.

## 1. Introduction

Defects created after curettage of benign bone tumors may be filled with autologous, allograft, or synthetic graft materials [1, 2]. In our prior use of the synthetic bone graft substitute ultraporous beta-tricalcium phosphate (TCP), the material performed well with the addition of local blood alone, but there was radiographic evidence of graft material that had not fully incorporated after a year or longer [3]. Although prolonged persistence of unincorporated TCP may serve as a potential stress riser, no adverse effects were observed in that study, and subsequent reports on its use have also been promising [4–6].

The addition of bone marrow aspirates to the TCP material has been suggested to enhance incorporation. In our completed randomized prospective clinical study, we were unable to show a significant difference in radiographic incorporation for TCP combined with bone marrow aspirate (TCP-BMA) compared to TCP alone [4]. However, both groups showed qualitative evidence of progressive TCP incorporation over time. No quantitative analysis of the CT scans from that study has been reported.

Despite the potential of allowing more detailed analysis of bone defect healing, CT scans have been underutilized for assessing the incorporation of graft materials. Most of the recent usage has been in the areas of spine, periodontal, and foot/ankle procedures [5–16]. The only bone defect study in which CT scans have been utilized is the aforementioned prospective clinical evaluation of TCP, but the analysis was performed in an arguably subjective fashion by radiology interpretation [4]. No detailed quantitative CT analysis was performed.

The purpose of this study was to use a quantitative CT-based volumetric imaging analysis approach for a detailed quantitative subanalysis of the previously reported larger prospective study comparing the use of TCP alone to TCP with BMA as bone fillers after curettage of benign lesions. Our hypotheses were that TCP incorporation into new bone would proceed at the greatest rate in (1) smaller defects in smaller bones, (2) younger patients, and (3) those that received BMA and that (4) evidence of* cortical* bone healing would be identified beyond filling of the* medullary *defect.

## 2. Methods

Under an IRB-approved prospective randomized controlled clinical trial design study, patients (age 4-65) undergoing surgery by the first author (TAD) at a single institution (SUNY Upstate Medical University) to remove benign bone lesions via curettage were randomized to two treatment groups. Accrual began in 2004 and was all follow-up was completed during calendar year 2009. Patients with active infection, bone marrow disorders, other contraindications to use of supplemental bone marrow aspiration, those with posttraumatic defects, those who preferred autologous or allogeneic graft material alone (without synthetic filler) and those who declined follow-up were excluded. One group underwent curettage and grafting with ultraporous beta tricalcium phosphate (TCP, Vitoss, Orthovita, Inc.) alone and the other group underwent curettage and grafting with TCP and BMA (TCP/BMA) [4].

For the purposes of this study, the CT scans provided the outcome variables based upon the mentioned quantitative CT-based volumetric imaging analysis. Of the 55 patients who completed the randomized prospective trial, CT scans were obtained in 34 patients at approximately one year postoperatively. Five of those patients also had CT scans at 2 years and 1 patient at 3 years. However, due to the transition to completely digital imaging over the time course of this study, twenty patients (11 male / 9 female) with twenty-one lesions had one or more CT scans available for digital analysis (TCP=10, TCP/BMA =11 lesions) and form the basis of this study. Clearly, potential bias is introduced by the exclusion of those CT scans that were unable to be retrieved for digital analysis, but without access, the quantitative analysis was simply not able to be performed. In order to minimize the potential for additional bias, all remaining subjects were included for analysis.

During surgery, the lesion was first curetted completely. In cases of more aggressive lesions, extended curettage was accomplished, consisting of extension of the intralesional margin by high speed burr in all cases and use of adjunctive argon beam coagulation in some. During curettage, decompression of the proximal and/or distal medullary cavity, where feasible, was done in order to ensure that the lesion was eliminated and to enhance healing. Morsellized grains of TCP were compacted into the defect to fill it. The graft material was impacted with bone tamps into the periphery of the defect until it was firmly compacted. Then, more graft was added and impacted in an identical fashion. This was done until the defects were completely filled and the surface of the graft material was flush with the surface of the cortical bone overlying the defect. Those in the TCP/BMA group underwent the same procedure but also underwent bone marrow aspiration. The bone marrow aspirate was harvested with a standard bone marrow aspiration needle from the anterior iliac crest in volumes of 2-3 cc aliquots per pass. The aspiration needle was repositioned through a single point of insertion in the skin for each pass within the ilium. The ratio of BMA to TCP ranged from 1:3 to 1:2 [4]. The bone marrow was mixed with the TCP before the insertion into the defect in an identical fashion as previously described. Prophylactic internal fixation was performed to maintain bone stability and minimize the risk of fracture during defect healing in six of the 21 lesions.

Each patient was managed using the same postoperative guidelines based on anatomic location, size and characteristics of the lesion. For subjects with upper extremity lesions, upper body activities including but not limited to throwing, lifting, and strength training were restricted for 6 weeks postoperatively and until the patient lacked noticeable tenderness or pain in addition to radiographic evidence of some healing of the defect. Most of the patients with lesions in the lower extremity were asked to be toe-touch weight-bearing with axillary crutches for the first 6 weeks postoperatively. This was followed by slow resumption of complete weight bearing activities once the pain and tenderness subsided and radiographic analysis revealed signs of healing. Lower extremity lesion patients were allowed to return to usual activities, including impact activities, 3 months postoperatively under normal circumstances.

CT scans were obtained at approximately 1 year using a Siemens Sensation 16 scanner (Siemens AG, Erlnagen, Germany). For five patients, a second series of scans were obtained at approximately 2 years postop for clinical follow-up purposes. In one case, a third scan was obtained at 3 years postop, also for clinical reasons. The CT scan protocols were based on standard of care for the particular skeletal location and ranged from 100 to 120 kV (median: 120 kV) with 33 to 198 mAs (median: 91mAs), inplane resolution of 0.17 to 0.91mm (median: 0.48mm), and scan spacing of 0.7 to 1.0mm (median: 0.8mm). Slice thickness was 1mm for all scans.

**Figure 1:**
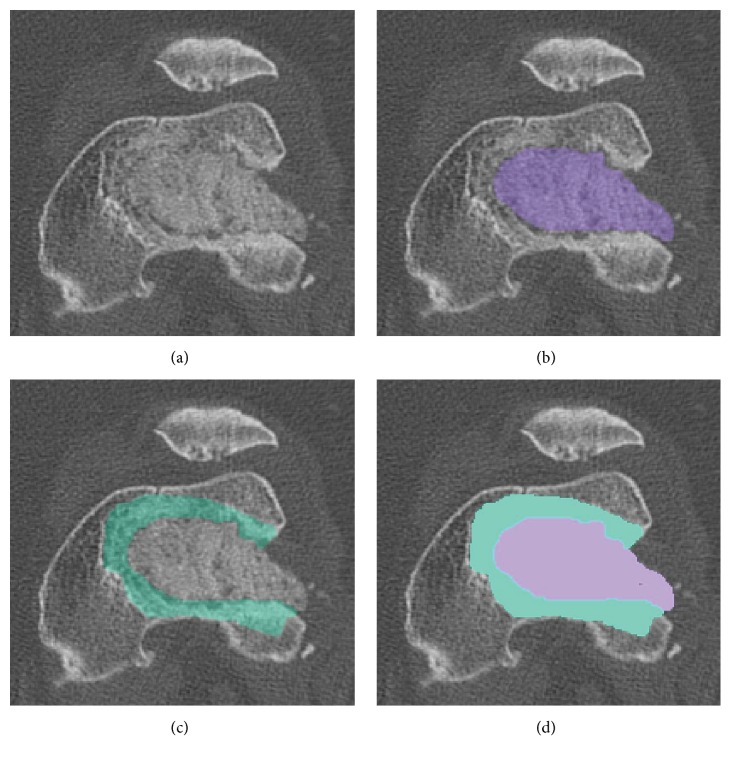
Axial section of distal femur with defect filled by TCP (a). The mask of the TCP (b) and new bone (c) are identified and combined (d).

**Figure 2:**
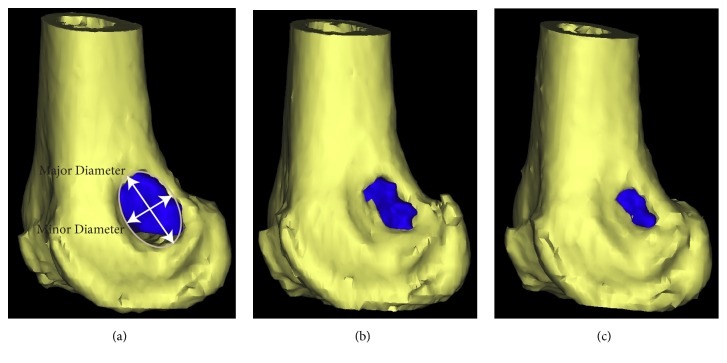
A 3D reconstruction of the example shown in Figure 1 at 1 year (a), 2 years, (b) and 3 years (c) postoperatively. The TCP is shown in dark blue. The new bone formation is not shown for clarity. The major and minor diameter of the cortical defect are shown (a).

**Figure 3:**
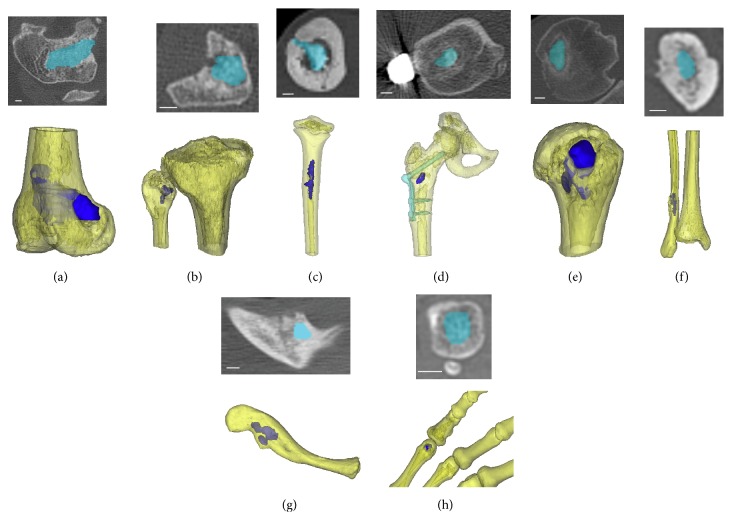
Representative axial CT and 3D reconstructed images of remaining TCP (blue) at 1-year postop for distal femur (a), proximal fibula (b), proximal tibia (c), proximal femur (d), proximal humerus (e), distal fibula (f), clavicle (g), and metacarpal (h).

Quantitative CT-based volumetric imaging analysis was as follows. Digital image sets were imported into MIMICS image processing software (Materialise, Plymouth, MI) to identify and segment the defect region into un-remodeled TCP graft material (TCP), newly formed bone tissue (NB), and the surrounding original bone (Figure 1). TCP was distinguished by high radiodensity compared to surrounding bone. The new bone (NB), occupying space between the original bone and TCP, was distinguished by the bony margin between new and existing bone. Masks were created for these three regions using a combination of gray scale thresholding and manual identification (Figure 1). The masked regions were built into three-dimensional volumetric objects from which the volume (cc) of TCP remaining (TCP) and TCP incorporated into new bone (NB) growth was determined (Figures 2 and 3). The total volume of the original defect (DV) was assumed to be the sum of these two values (NB + TCP). The new bone fraction (NB/DV) was used to assess TCP graft incorporation.

**Figure 4:**
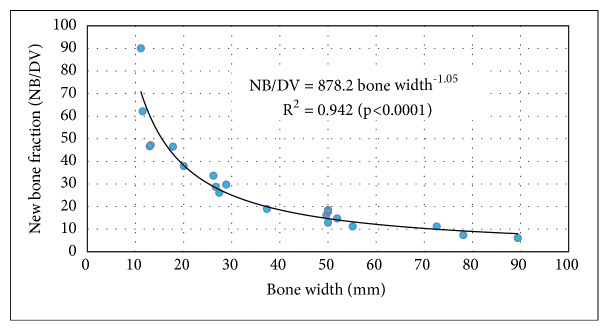
Initial defect volume (cc) as a function of bone width.

Bone width (mm) was measured at the midpoint of the defect. This was measured medial-lateral in the coronal plane at the proximal-distal midpoint for long bones. In the clavicle, it was measured cephalocaudad in the coronal plane located at the midportion of the lesion mediolateral. As expected, DV was closely related to size of the bone. (Figure 4)

The size of the remaining cortical defect was determined by fitting an ellipse to the cortical defect in the 3D image and measuring the lengths of the major and minor axis. The average of the major and minor axis lengths was divided by the bone diameter in the region, resulting in the cortical defect fraction (CDF).

To determine if TCP incorporation was greatest in smaller defects at one year after surgery, a linear regression model was used with new bone fraction (NB/DV) as the dependent variable and original defect volume (DV) as the independent variable. To determine if TCP incorporation was related to the size of the bone being grafted, another linear regression model was used with NB/DV as the dependent variable and bone width as the independent variable. To determine if incorporation of TCP into new bone would be greater in younger patients, a linear regression model was used with the new bone (NB) as the dependent variable, and age, DV, and the interaction between age and DV (age*∗*DV) as independent variables. To determine if the amount of new bone was dependent on use of BMA, an analysis of covariance was used with use of BMA as an independent variable, and DV as the covariate.

## 3. Results

Participants had a mean age of 22 years with a range from 4 to 66 years at time of 1 year CT. Nine patients (ten lesions) had been randomized to receive TCP alone while 11 patients (11 lesions) had been randomized to receive TCP with BMA. The 21 bone lesions were located in the proximal femur (N=4), distal femur (N=5), fibula (N=3), tibia (N=2), humerus (N=2), clavicle (N=2), metacarpal (N=1), proximal phalanx (N=1), and middle phalanx (N=1). The two phalangeal lesions were from the same patient.

Summary statistics for the 21 skeletal defects are shown in Table 1. Defect volume ranged from 0.12 cc to almost 100 cc with median volume of 8 cc. New bone fractions (NB/DV) ranged from 0.54 to 1.0 with a mean of 0.76, indicating that on average 76% of the defect volume was replaced with new bone. The cortical defect fraction (CDF) ranged from 0 to 0.67 with a median of 0.15. Five of the defects had no remaining cortical defect at 1 year.

**Figure 5:**
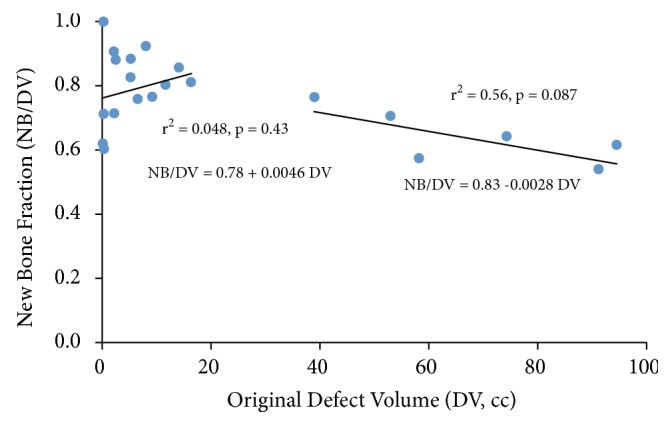
The new bone fraction (NB/DV) was greater for small defect volumes (DV). Overall, there was a significant decrease (p=0.0029) in NB/DV with increasing DV (r^2^=0.38). When separated into smaller (<20cc) and larger defect volume groups (>20cc), there was a trend towards decreased new bone fraction in the >20cc group.

Analyzed overall, there was significant (p=0.0029) negative correlation (r^2^ = 0.38) between the fraction of new bone (NB/DV) and the original defect volume (DV) (Figure 5). However, when defects less than and greater than 20 cc size were analyzed separately, this relationship was not quite as clear. The same inverse linear relationship between NB/DV and DV held true for DV greater than 20 cc with an even stronger correlation coefficient (r^2^=0.56), although the fewer numbers (n = 6) in this subset analysis did not allow statistical significance (p = 0.09). Although in the subset analysis for defects less than 20 cc there appeared to be a positive relationship between NB/DV and DV, the relationship was weak and not significant (r^2^ = 0.048, p = 0.43). The large amount of scatter in the NB/DV for the small defects is due to the division between two small numbers as DV approaches zero. For the remainder of the analyses, new bone volume (NB) was used as the primary dependent variable.

**Figure 6:**
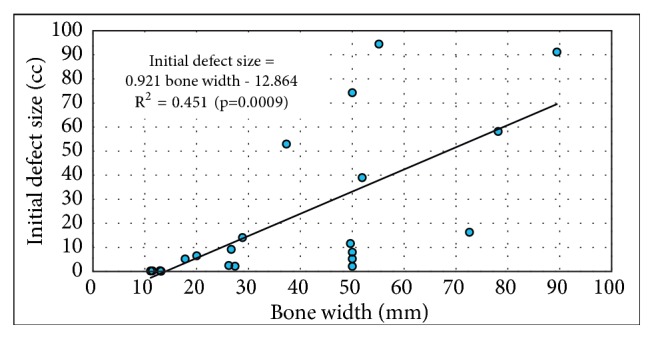
New bone fraction (NB/DV) was inversely proportional to bone width.

In part likely due to the linear relationship between defect size and bone size (Figure 4), NB/DV was highly related to the bone size (Figure 6). The largest lesions were in the distal femurs and proximal tibias, and the smallest lesions were in the phalanges, metacarpal, and clavicle.

**Figure 7:**
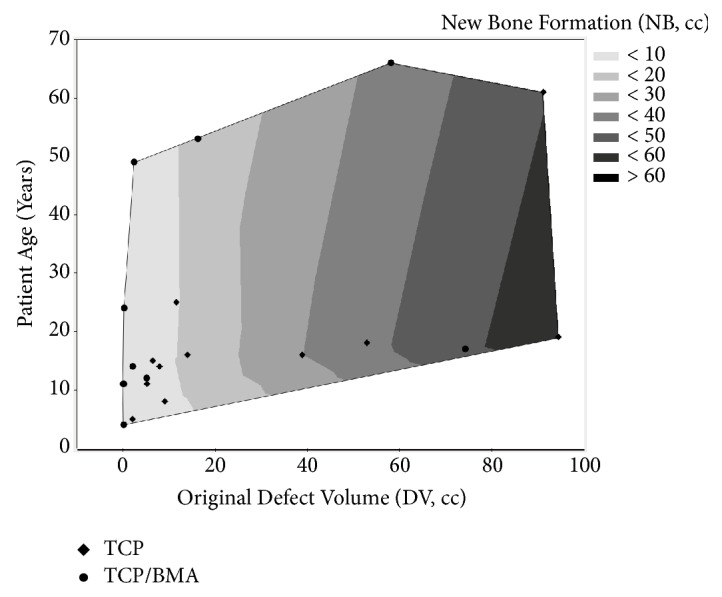
A contour plot of the influence of patient age and original defect volume (DV) on new bone formation (NB). Using a multiple regression model, patients with larger DV had higher NB (p<0.0001). Increasing age coupled to increasing DV (age*∗*DV) had reduced NB (p=0.0003). NB is TCP/BMA data (diamonds) and TCP data (filled circles) are shown.

For larger defect volumes, younger patients were more successful at incorporation (Figure 7). Using a linear regression model, the defect volume (p<0.0001) was a strong positive predictor, and the interaction term (Age*∗*DV) (p=0.0003) was a strong negative predictor of new bone formation (or incorporation). Age itself was not a significant factor (p=0.50).

**Figure 8:**
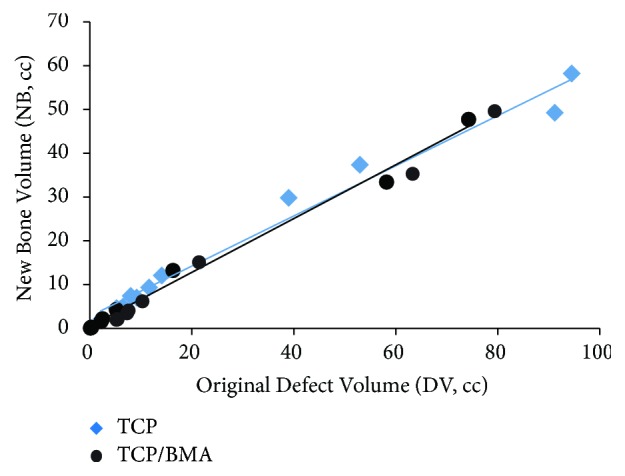
New bone volume (NB) as a function of original defect volume (DV) for the TCP and TCP/BMA groups. Linear regression lines through the two groups are also shown.

There was no statistically significant difference in new bone formation (NB) between TCP alone and TCP with BMA groups (p=0.27). Additionally, the slope of the NB versus DV relationship was not different for the two groups (p=0.29) (Figure 8). The two groups were not significantly different with respect to fractional cortical defect ratio (CDF) (p=0.576).

**Figure 9:**
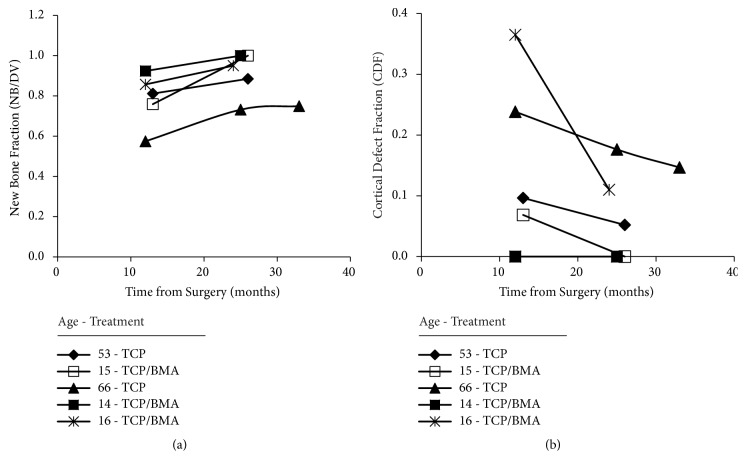
Medullary new bone fraction (a) and cortical defect fraction (B) as a function of months of healing for the five patients with more than one serial CT scan. Note that progressive improvement in medullary NB formation (incorporation) occurred in all of the five patients (a) and progressive decrease in cortical defect fraction (defect graft incorporation) occurred in four out of the five patients (b).

Evidence of cortical bone healing identified beyond filling of the medullary defect was impossible to evaluate in subjects with only one CT scan. However, in the anecdotal and very small subset of five patients with CT scans at a second and third time point, qualitative evidence suggesting progressive medullary new bone formation and cortical bone healing was observed (Figure 9).

## 4. Conclusions

Synthetic alternatives to autogenous and allogeneic bone graft fillers continue to be developed and explored [1, 2, 17–19]. Variability in rates of graft incorporation exist between materials, with calcium sulfate products thought to resorb rather quickly and hydroxyapatite to persist indefinitely [3, 19–22]. Tricalcium phosphate has an intermediate pattern of graft incorporation, which has led to frequent clinical usage [1–12, 14–16, 23]. In our completed prospective clinical trial from which the CT scans in this study were derived, no significant difference was detected in bone graft incorporation parameters between patients with benign curetted defects randomized to receive either ultraporous beta-TCP alone or combined with autogenous iliac crest bone marrow [4]. Numerous additional clinical studies have evaluated the use of ultraporous beta-TCP for other indications, including trauma and spinal fusion [7, 9–12, 14–18, 24–29]. None have established clearly whether there is a benefit to the addition of BMA to the TCP. However, none have utilized the more detailed quantitative CT based volumetric analysis described herein.

The main impetus behind the prospective randomized study had been to determine if the addition of bone marrow to ultraporous beta-TCP would improve parameters of graft incorporation [4]. A prior retrospective study suggested that use of this graft material mixed with only local blood incorporated more slowly than was desirable [3]. Since prolonged graft persistence of other graft materials, such as hydroxyapatite, served as stress risers and resulted in late fractures, the addition of bone marrow was hypothesized to improve graft incorporation, as it has been shown to do in the laboratory [30–32]. However, neither the previously reported study with qualitative radiographic analysis nor the current quantitative CT analysis based results support this hypothesis [4]. Potential explanations for this lack of difference, apart from simple lack of a true difference, are numerous. First, the number of CT scans available for the current analysis were much smaller even than those available for the larger parent study due to a transition to digital images. Second, the surgical technique used for the study may have contributed to blurring the differences. In all study patients the adjacent intramedullary canal was penetrated surgically in order to allow access to adjacent bone marrow. Additionally, the amount of bone marrow aspirate may have been suboptimal to afford a healing advantage to the bone marrow group [4].

With respect to whether quantitative analysis would be able to delineate differences in graft incorporation between groups suggested by earlier nonquantitative studies to be different (e.g., age and size of the defect), the results were more fruitful but complex. Based on the current results, graft incorporation was correlated in part with the size of the original curetted defect. Overall, this was a significant linear inverse relationship, but when separated out into a subset analysis of defects larger than or smaller than 20 cc, only the larger defects held true to this relationship. Even more notable than the relationship with defect size was the strong negative relationship of graft incorporation to bone width (and hence medullary bone size). Defects in larger bones had lower graft incorporation in this CT based analysis. On one hand, this may seem counterintuitive if one theorizes that a larger medullary surface area provides more medullary cancellous bone and vascularity to stimulate healing. On the other hand, if the bone graft incorporates by creeping substitution at a relatively linear rate, as it seems to in the presented cases, the absolute size of the defect would mean that indeed smaller bones with smaller defects would result in a high fraction of bone healing at any given time postoperatively.

The relationship with age was complex and closely tied to defect volume. For larger defect volumes, younger patients were more successful at graft incorporation. Although age itself was not an independently significant factor in our regression model, as defect volume increased, advanced age had more of a negative impact on new bone formation.

This series is unable to provide definitive evidence of cortical healing with Vitoss as only five patients had serial CT scans. However, on those scans we did observe gradual healing over time. Based on the limited subset analysis, this evidence has to be considered anecdotal at best.

One finding that appears to contradict reports in the literature is that for many of the currently reported lesions, graft incorporation remained incomplete, even after one year of follow-up. Others have reported complete bone graft incorporation in between 6 and 9 months with ultraporous beta-TCP or similar beta-TCP compounds, but these reports have been based upon qualitative means of evaluation rather than CT [5, 6]. The current quantitative analysis and CT images also clearly show that the graft incorporation and trabeculation occurs from the periphery to the center, in a centripetal fashion, rather than the reverse, as has been suggested [6].

Limitations of this study include use of small amounts of bone marrow relative to the TCP graft material, poor statistical power, loss of ability to analyze some early study images collected prior to current digital imaging techniques, and presence of hardware artifact in patients who had simultaneous internal fixation. The ultraporous beta-TCP product used in this study has been recommended to be used in a 1:1 ratio of bone marrow to TCP, far greater than the ratio of between 1:2 and 1:3 employed in this study. The original power analysis for this study suggested an 80% power to show a 25% difference with the number of patients enrolled, but our attrition rate for the CT was 39%, hampering our statistical power. Weaknesses acknowledged, this study remains unique in its method of utilizing quantitative volumetric CT analysis of bone graft incorporation. Future study designs evaluating efficacy of bone grafting materials should consider inclusion of CT analysis of this sort in order to better elucidate the many remaining questions surrounding graft incorporation.

## Figures and Tables

**Table 1:** Descriptive statistics for 21 bone lesions.

Parameter	Mean	Median	Std Dev	Min	Max
Patient Age	22.3	16	18.3	4	66

Original Defect Volume (DV, mm^3^)	23520	8006	31390	107	94490

New Bone Volume (NB, mm^3^)	15480	7025	18780	66	58220

Remaining TCP Volume (mm^3^)	8037	1570	13010	0	41910

New Bone Fraction (NB/DV)	0.76	0.76	0.13	0.54	1.0

Cortical Defect Diameter Major (mm)	11.2	7.7	15.0	0	62.0

Cortical Defect Diameter Minor (mm)	5.4	2.2	6.7	0	22.2

Cortical Defect Fraction (CDF)	0.21	0.15	0.20	0	0.67

## Data Availability

The data used to support the findings of this study are available from the corresponding author upon request.
